# Loss of Circulating Exosomal miR-92b is a Novel Biomarker of Colorectal Cancer at Early Stage

**DOI:** 10.7150/ijms.34540

**Published:** 2019-08-14

**Authors:** Li Min, Lei Chen, Si Liu, Yang Yu, Qingdong Guo, Peng Li, Shengtao Zhu

**Affiliations:** Department of Gastroenterology, Beijing Friendship Hospital, Capital Medical University, National Clinical Research Center for Digestive Disease, Beijing Digestive Disease Center, Beijing Key Laboratory for Precancerous Lesion of Digestive Disease. No.95, Yong'an Rd, Xicheng District, Beijing,100050, P. R. China.

**Keywords:** exosome, miR-92b, colorectal cancer, early diagnosis, biomarker.

## Abstract

Early diagnosis of colorectal cancer (CRC) is clinically critical but technically challenging, especially in a minimal-invasive way. Emerging evidence suggests that exosome-encapsulated microRNAs (miRNAs) is a kind of promising cancer biomarker. Here we investigated the predictive potential of exosomal miR-92b in plasma samples obtained from 114 participants [40 CRC, 22 colorectal adenomas (CA), 52 non-neoplasm controls (NC)] by RT-qPCR. We found that exosomal miR-92b level was significantly down-regulated in CRC patients compared with CA and NC patients, especially in CRC at stage II, regardless of lymph node metastasis and invasive depth. The AUC in distinguishing CRC, CA and NC from each other ranged from 0.631 to 0.793, while a higher AUC of 0.830 was achieved in differentiating CRC at clinical stage II/III from NC individuals. Additionally, a logistic model integrating miR-92b with age showed a significantly improved accuracy in distinguishing CRC patients from NC (AUC increased from 0.793 to 0.867). Taken together, our findings indicated that decreased expression of exosome-derived miR-92b in plasma is a promising biomarker for early detection of CRC.

## Introduction

Colorectal cancer (CRC) is the third most common cancer in men and second in women with 1,360,000 new cases occurred worldwide, which is also one of the leading causes of malignancy with 694,000 death in 2012^1^. The 5-year survival rate for CRC patients is predominantly depended on different tumor stages with approximately 90% at an early stage but <10% at a metastatic stage^2^. Therefore, target population screening, early diagnosis, and timely treatment are essential for high-risk individuals. Circulating biomarkers emerge as a class of minimally invasive approaches in early detection and prevention of CRC during the past decade. Carcinoembryonic antigen (CEA) and carbohydrate antigen 19-9 (CA19-9) which have been generally used in diagnosis of CRC and other types of cancer, exhibited a less-than-desirable sensitivity and specificity^3^-^5^. Thus, identifying novel CRC-specific biomarkers is of great importance for early detection of CRC, especially for patients at curable stages.

MicroRNAs (miRNAs) are endogenously derived non-coding RNAs with 19-25 nucleotides discovered in various organisms and critically involved in gene regulation^6^. Increasing studies have revealed that although miRNAs do not encode any proteins, they post-transcriptionally interfere with both the stability and translation of protein-coding mRNAs, resulting in dysfunction of cell proliferation, apoptosis, metastasis as well as tumorigenesis and progression^7^. However, the exact biological function of miRNAs is complicated. Both oncogenic and tumor-suppressive roles of different miRNAs, or even the same miRNA, have been reported in various types of cancer 8-10. MiR-92b is a *Janus-faced* cancer-associated miRNA, which was initially reported being overexpressed in primary brain tumor^11^. After that, several studies further clarified its oncogenic regulatory role in glioblastomas^12^, ^13^, non-small cell lung cancer (NSCLC)^14^, ^15^, bladder cancer^16^ and osteosarcoma^17^. On the contrary, the tumor-suppressive role of miR-92b in many gastrointestinal cancers, such as pancreatic cancer^18^, esophageal squamous cell carcinoma (ESCC)^19^, was also revealed*.*

Exosomes are 30-100nm extracellular vesicles secreted by multiple types of cells and are released into a variety of body fluids. Exosomes consist of nucleotides (such as miRNA), proteins, lipid and so on, participating in signal transmission and cell-to-cell communication^20^, ^21^. MiRNAs in exosomes could regulate tumor cell proliferation, angiogenesis and invasion^22^-^24^, which have been considered as one of the most effective biomarkers for disease diagnosis in the past few years. Additionally, exosome-encapsulated miRNAs exhibit better stability compared with circulating free miRNAs since such membrane vesicles may serve as shelters, protecting inner miRNAs from endogenous RNase activity^25^-^27^. Thus, exosome-derived miRNAs would be a much more promising and robust biomarker type in cancer diagnosis.

Here we evaluated the circulating exosomal miR-92b level of CRC patients, high-risk individuals with precancerous lesions, and healthy controls, which would reveal the clinicopathological significance of exosomal miR-92b in plasma and its potential application as a liquid biopsy tool for CRC screening.

## Materials and Methods

### Patients and clinical samples

All peripheral blood samples were obtained from patients who underwent endoscopic submucosal dissection at Beijing Friendship Hospital, Capital Medical University from January 2017 to June 2018. The study was approved by the ethics committee of Beijing Friendship Hospital and written informed consent was obtained from each patient. Finally, we included a total of 114 plasma samples from patients with CRC (n=40), colorectal adenoma (CA, n=22) and other non-cancerous lesions (NC, n=52). Each blood sample was firstly centrifugated at 3,000 ×g for 15 min at 4 °C. Then the plasma was aspirated and stored at -80 °C before use. Clinical stage was classified using the TNM system of classification. All relevant data including age, gender, tumor size, invasive depth, tumor location, lymph node metastasis, Yamada subtype, Paris subtype for each sample were recorded from our clinicopathological database. Details of clinical characteristics of all cases included were listed in Table 1.

### Plasma exosome isolation

We followed ultracentrifugation procedure for plasma exosome purification according to previous studies^28^, ^29^. In brief, plasma samples were firstly centrifugated at 3,000 ×g for 15 min to remove cell debris. The supernatant was diluted eight times with PBS (phosphate-buffered saline), followed by centrifugation at 13,000 ×g for 30 min and filtration through a 0.22μm filter to remove large particles. Then the supernatant was ultracentrifugated at 150,000 ×g, 4 °C for 4h and the pellets were washed with PBS.

### Transmission Electron Microscopy (TEM)

The isolated exosomes were re-suspended in PBS and 20 µl of the suspension was placed on a carbon-coated copper grid and incubated together for 10min at room temperature. Next, the grid was washed by sterile distilled water and then put in contact with 2% uranyl-oxalate solution for 1min and dried for several minutes. Finally, the grid was observed using an electron microscope (JEOL-JEM1400, Tokyo, Japan).

### Nanoparticle tracking analysis (NTA)

In order to identify the exact size and quantity of isolated particles, the suspension with concentration between 1x10^7^/ml and 1x10^9^/ml was examined using ZetaView PMX 110 (Particle Metrix, Meerbusch, Germany) equipped with a 405nm laser. A video of 60-sec duration was taken with a frame rate of 30 frames/sec, and particle movement was analyzed using NTA software (ZetaView 8.02.28).

### Western blot analysis

The suspension mentioned above was diluted with 5X sodium dodecyl sulfonate (SDS) buffer and was boiled for 10mins, in preparation for the following western blot analysis (10% SDS-polyacrylamide gel electrophoresis; 50 µg protein/lane) of two positive markers (CD63 and TSG101) and one negative marker (calnexin) of extracellular vesicles. Rabbit polyclonal antibody CD63 (sc-5275, Santa Cruz, CA, USA), TSG101 (sc-13611, Santa Cruz, CA, USA) and calnexin (10427-2-AP, Promega, Madison, WI) were used. The protein bands were detected using an enhanced chemiluminescence system (Bio-Rad, USA).

### Extraction of Total RNAs from Plasma Exosomes

Total RNAs of exosomes were extracted using miRNeasy® Mini kit (Qiagen, cat. No. 217004) according to the manufacturer's protocol. RNA quality (including degradation, contamination, concentration, purity and integrity) was accessed by 1.5% agarose gel electrophoresis combined with RNA Nano 6000 Assay Kit of the Agilent Bioanalyzer 2100 System (Agilent Technologies, CA, USA).

### RT-qPCR for miRNAs of Plasma Exosomes

MiR-92b expression of plasma exosomes was quantified by RT-qPCR. TaqMan^™^ advanced miRNA assays were performed for miRNA quantification using Life TaqMan Advanced miRNA cDNA Synthesis Kit (Life Tech, Carlsbad, CA, cat. A28007) and Life TaqMan Fast Advanced Master Mix (Life Tech, Carlsbad, CA, cat.4444557). The same amount of *Caenorhabditis elegans* cel-39-3p miRNA was mixed into each exosome sample as an external control during the whole process. Besides, a specific probe for miR-92b (cat. 479207, Life Technologies, Carlsbad, USA), cDNA template of each sample and TaqMan Universal PCR Master Mix (Life Technologies) were applied for RT-qPCR procedure using ABI7500 qPCR system (Applied Biosystems) following the manufacturer's protocol. Relative quantification of miRNA expression was calculated using 2 ^-ΔΔCT^ method.

### Statistical analysis

Data are expressed as mean ± SD. The relationships between miRNA expression and clinicopathological factors were analyzed using independent-samples T test, Wilcoxon test and Kruskal-Wallis test. All tests were two-tailed and False Discovery Rate (FDR) was controlled for multiple comparisons. P<0.05 was considered statistically significant. Diagnostic accuracy was assessed by receiver operating characteristic (ROC) curves analysis, and the area under the ROC curve (AUC) was also calculated. Packages plyr and reshape2 were used for data sorting and restructuring, while ggplot2 was used for visualization of results.

## Results

### Identification of exosomes isolated from plasma

**Figure 1:**
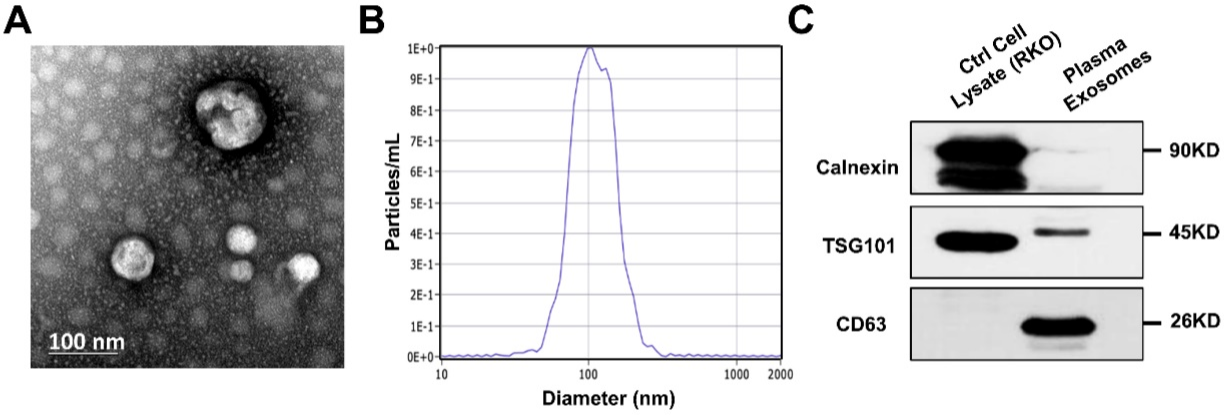
** Identification of exosomes isolated from patients' plasma.** (A) Scanning transmission electron microscopy (TEM) revealed the external features of the exosomes isolated from plasma. The exosomes were oval or bowl-shaped capsules without nucleus. (D) NTA demonstrated that the exosomes isolated from patients' plasma were 75-200nm in diameter. (C) Characteristic markers of extracellular vesicles were verified by Western Blot. Enrichment of two positive markers (TSG101 and CD63) were detected while the negative marker (calnexin) was absent in the isolated exosomal samples.

To verify the effectiveness of ultracentrifugation method for exosome isolation, we examined the nanoparticles by TEM and NTA, along with two positive and one negative markers of extracellular vesicle. We captured images of oval or bowl-shaped microvesicles (Fig. 1A), with diameters mostly ranging from 75nm to 200nm (Fig. 1B). Enrichment of two positive markers (TSG101 and CD63) was both detected while the negative marker (calnexin) was absent (Fig. 1C). Thus, the purification and integrity of isolated exosomes were confirmed.

### Plasma exosome-derived miR-92b level in CA and CRC of different stages

**Figure 2:**
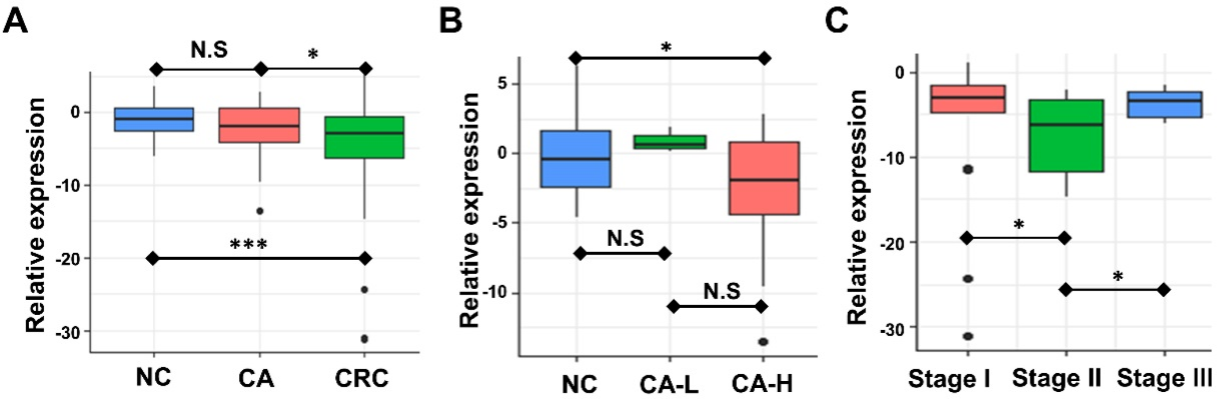
** Association between circulating exosomal miR-92b level and CRC stage.** (A) The expression of exosomal miR-92b in plasma was significantly down-regulated in CRC patients compared with both CA and NC patients* (p=0.019, p<0.001, respectively)*. No statistical significance was exhibited between the CA and NC group(*p=0.164*). (B) The circulating exosomal miR-92b level was much lower among CA-H patients than that in NC patients (*p=0.035*). No statistical significance was observed between CA-H and CA-L groups and between CA-L and NC groups (*p=0.180, p=0.208, respectively*). (C) CRC patients at TNM stage II obtained the most decreased level of exosome-derived miR-92b in plasma compared to those who with TNM stage I and III (*p=0.030, p=0.015, respectively*).

We examined the circulating exosomal miR-92b level of CRC, CA, and NC participants. Expression of miR-92b was assessed by RT-qPCR. Fig. 2A showed that the circulating exosomal miR-92b level of CRC patients was significantly lower than that of both the CA and NC participants (*p=0.019, p<0.001,* respectively). However, no statistical significance was observed between the CA and NC group (*p=0.164*). Thus, exosomal miR-92b in plasma could be a potential biomarker to distinguish CRC from CA and NC, but could not distinguish CA from NC.

We further investigated the potential change of plasma exosome-derived miR-92b level among CA patients in different stages. Exosomal miR-92b levels was significantly decreased in CA patients with high-grade intraepithelial neoplasia (CA-H) compared with NC individuals, but no significant change was observed in CA patients with low-grade intraepithelial neoplasia (CA-L) compared with NC (Fig. 2B, *p=0.035, p=0.180,* respectively). Thus, exosomal miR-92b in plasma could be a potential biomarker to distinguish CA-H and NC, but could not distinguish CA-L from NC.

Additionally, the potential change of plasma exosome-derived miR-92b level among CRC patients of different stages was also evaluated. Interestingly, plasma exosome-derived miR-92b level in patients at TNM stage II decreased compared to both stage I and stage III patients (Fig. 2C, *p=0.030, p=0.015,* respectively). Combining with the results of NC and CA individuals, we suggested that the decrease of exosomal miR-92b in plasma mainly occurs during the pathophysiological process from colorectal adenoma CA-L to stage II carcinoma.

### Association between plasma exosome-derived miR-92b and CRC clinical factors

We further investigated the potential association between plasma exosome-derived miR-92b and other clinicopathological characteristics. A little but not significant up-regulation of miR-92b in patients with tumor size >2cm was observed (Fig. S1A, *p=0.744*) was observed. There was no statistically significant change among patients with different tumor location (Fig. S1B, Rectum, Left colon *v.s.* Right colon,* p=0.285, p=0.950,* respectively), different Yamada subtype (Fig. S1C, subtype I, II and III* v.s.* subtype IV,* p=1.000, p=0.192*, *p=0.355,* respectively). Besides, none of other included factors, such as age, sex, invasive depth and lymph node metastasis, was significantly associated with miR-92b level either. (Fig. S1D-G). We also evaluated the correlation between exosomal miR-92b level and clinicopathological characteristics in patients with non-cancerous lesions of CA and NC groups (Fig. S2A-E).

### Evaluation of exosomal miR-92b in plasma as a predictive biomarker for CRC

**Figure 3:**
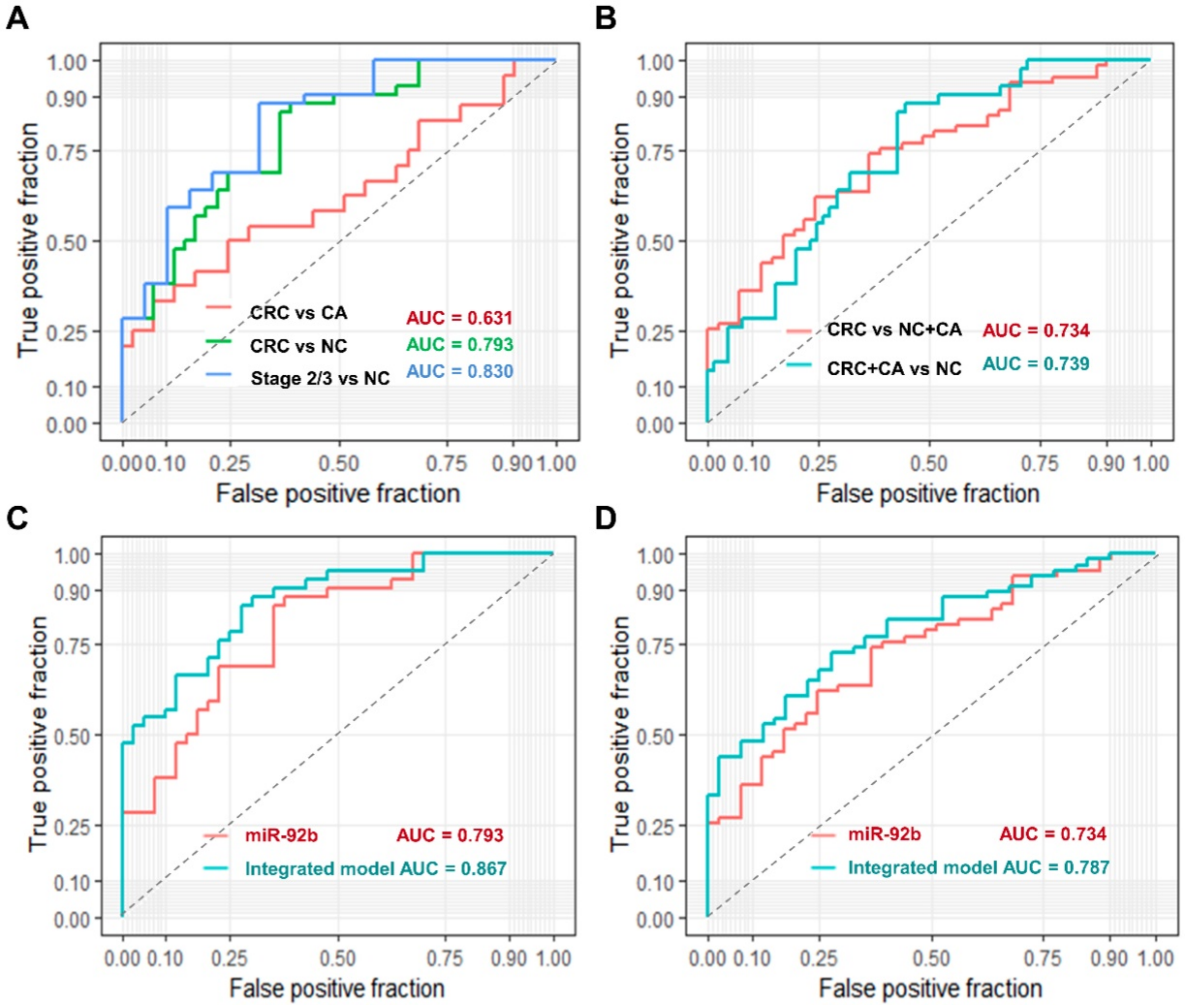
** Diagnostic power of plasma exosomal miR-92b as a biomarker of CRC.** (AB) Verification of exosomal miR-92b as biomarkers in each tumor stage of CRC. The AUC in distinguishing CRC from BC, or NC, or BC+NC was 0.631, 0.793 and 0.734, respectively, while the AUC of 0.739 was exhibited in distinguishing CRC and CA from NC patients. The best AUC of 0.830 was obtained in differentiating CRC at clinical stage II or III from NC ones. (CD) Verification of the integrated biomarker panel (miR-92b level plus age) among CRC patients. The AUC of the combined biomarker increased to 0.867 and 0.787 in distinguishing early CRC from NC and in differentiating CRC from NC and CA individuals, respectively, in comparison of using exosomal miR-92b alone (0.793 and 0.734, respectively).

We used ROC curve analysis to evaluate the predictive value of circulating exosomal miR-92b. Overall, exosomal miR-92b in plasma showed a promising potential in distinguishing CRC patients (AUC = 0.793), especially stage II/III CRC patients (AUC = 0.830) from NC individuals, but its ability in distinguishing CRC patients from CA individuals is not satisfying (AUC = 0.631, Fig. 3A).

Moreover, when we try to differentiate CRC patients from both CA and NC individuals, the AUC reached to 0.734 (Fig. 3B). Similar AUC level (0.739) was achieved when trying to differentiate CRC and CA patients from NC individuals (Fig. 3B). Additionally, we built a logistic model to distinguish CRC patients from NC individuals using exosomal miR-92b level and all clinical factors in a backward LR method. Only exosomal miR-92b level and age were included in the final model, which showed a much higher AUC than exosomal miR-92b itself (0.867 *v.s.* 0.793, Fig. 3C). A similar model integrated exosomal miR-92b level and age was also generated in the same way to distinguish CRC/CA patients from NC individuals (AUC=0.787, Fig. 3D). Therefore, the integrated model could serve as a promising diagnostic biomarker of CRC with high sensitivity and specificity.

## Discussion

Recently, a number of miRNAs have been reported to be stably detected in multiple body fluids such as plasma, serum, saliva and urine, suggesting their potential values as minimal-invasive circulating biomarkers of different cancers including CRC^30^-^32^. Moreover, it has been well informed that part of these circulating miRNAs are released from cancer cells by being capsuled in exosomes towards into body fluids and function as intercellular messengers^33^, ^34^. In this study, we aimed to evaluate a specific exosomal miRNA (miR-92b) in plasma as a liquid biopsy biomarker of CRC patients at different tumor stage.

We found that circulating exosomal miR-92b level was significantly decreased in CRC patients than that in healthy controls, especially those at TNM stage II. For patients with precancerous lesions, we only observed a reduced circulating exosomal miR-92b level in CA-H patients but not CA-L patients, suggested that the decrease of miR-92b occurred during the process from CA-L to CA-H. Additionally, we found that the circulating exosomal miR-92b level is relatively stable among patients with different tumor location, Yamada subtype, lymph node metastases. Thus, exosomal miR-92b level in plasma is resilient to disturbances caused by other clinical factors, which would render it the potential for a robust CRC biomarker.

Many researchers have reported on the potential of circulating plasma/serum miRNAs as both diagnostic and prognostic biomarkers for CRC patients^30^, ^32^, ^35^. Interestingly, different miRNAs may have opposite biological characteristics in different cancers, for example, functioning as oncogenes in one cancer type and as tumor-suppressors in another^36^. For miR-92b, its decrease in CRC plasma exosomes supported its tumor-suppressors in gastrointestinal cancers, which was reported in pancreatic cancer^18^, ESCC^19^, and *etc*.

Exosomes are enriched in the circulatory system and are able to protect their inner miRNAs from RNase degradation, suggesting that circulating exosome-encapsulated miRNAs are a much more robust diagnostic biomarker source than circulating free miRNAs due to their high stability and integrity. Additionally, considering that blood coagulation process would generate a lot of unexpected exosomes, we used plasma to conduct the extraction of exosomes instead of serum. Our study is the first to examine exosomal miR-92b expression in a medium sized population including CRC, CA, and NC. We found that exosomal miR-92b in plasma had a promising potential in distinguishing CRC patients from NC individuals (AUC = 0.793), but its ability in distinguishing CRC patients from CA individuals is relatively poor (AUC = 0.631). The results suggested that plasma exosomal miR-92b had a diagnostic power in distinguishing CRC but not CA from other non-neoplasms individuals.

Integrated diagnostic panels consisting of several combined factors have been previously reported as optimal models for cancers diagnosis^37^. Vychytilova-Faltejskova et al. established a four-miRNA signature including miR-23a, miR-27a, miR-142 and miR-376c with the AUC of 0.917 for early diagnosis of CRC^38^. In this study, the diagnostic accuracy was substantially improved in distinguishing CRC from NC patients when we integrated the patients' age (AUC increased from 0.793 to 0.867). We believed that the integrated predictive model may serve as a convenient and economic tool with both high sensitivity and specificity in clinical practice compared to previously reported diagnostic miRNA panels, considering that only one blood biological indicator need to be examined.

In conclusion, circulating exosomal miR-92b was significantly down-regulated in CRC patients, especially in those at stage II. Besides, a logistic model integrating miR-92b level with age obtained an increased diagnostic accuracy and may serve as a promising minimal-invasive tool for early CRC diagnosis.

## Supplementary Material

Supplementary figures and tables.

## Figures and Tables

**Table 1:** Clinical characteristics of all patients with CRC, CA and NC.

Factors	CRC (n=40)	CA (n=22)	NC (n=52)
**Age, n (%)**			
<55 years	4 (10.0)	6 (27.3)	22 (42.3)
≥55 years	36 (90.0)	16 (72.7)	30 (57.7)
**Gender, n (%)**			
Male	30 (75.0)	18 (81.8)	36 (69.2)
Female	10 (25.0)	4 (18.2)	16 (30.8)
**Tumor size, n (%)**			
<2cm	11 (27.5)	12 (54.5)	/
≥2cm	26 (65.0)	10 (45.5)	/
NA	3 (7.5)	0 (0.0)	/
**Invasive depth**			
Mucosa	14 (35.0)	22 (100.0)	**/**
Submucosa	25 (62.5)	0 (0.0)	**/**
NA	1 (2.5)	0 (0.0)	**/**
**Clinical stage, n (%)**			
I	22 (55.0)	**/**	**/**
II	9 (22.5)	**/**	**/**
III	6 (15.0)	**/**	**/**
NA	3 (7.5)	**/**	**/**
**Lymph node metastasis, n (%)**			
N	32 (80.0)	**/**	**/**
Y	7 (17.5)	**/**	**/**
NA	1 (2.5)	**/**	**/**
**Location**			
Right colon	7 (17.5)	4 (18.2)	/
Left colon	19 (47.5)	9 (40.9)	/
Rectum	13 (32.5)	6 (27.3)	/
NA	1 (2.5)	3 (13.6)	/
**Yamada subtype, n (%)**			
I	5 (12.5)	6 (27.3)	/
II	8 (20.0)	8 (36.3)	/
III	4 (10.0)	4 (18.2)	/
IV	2 (5.0)	4 (18.2)	/
NA	21 (52.5)	0 (0.0)	/
**Paris subtype, n (%)**			
Is	7 (17.5)	7 (31.8)	/
Ip	3 (7.5)	6 (27.3)	/
Isp	8 (20.0)	7 (31.8)	/
II	1 (2.5)	2 (9.1)	/
NA	21 (52.5)	0 (0.0)	/
**Tumor differentiation status, n (%)**			
CA-L	**/**	3 (13.6)	**/**
CA-H	**/**	16 (72.8)	**/**
SSA/NA	**/**	3 (13.6)	**/**
**Clinical diagnosis, n (%)**			
Gastritis	**/**	**/**	28 (53.8)
Cholelithiasis	**/**	**/**	4 (7.7)
PHT	**/**	**/**	4 (7.7)
Polyps	**/**	**/**	6 (11.6)
Others	**/**	**/**	10 (19.2)

*CRC,* Colorectal cancer; *CA,* colorectal adenoma; *NC,* non-cancerous lesion; *CA-L,* CA with low-grade intraepithelial neoplasia; *CA-H,* CA with high-grade intraepithelial neoplasia; *NA,* not available; *SSA,* sessile serrated adenoma; *PHT,* portal hypertension.

## Abbreviations

CRC: colorectal cancer
miRNAs: microRNAs
CA: colorectal adenomas
NC: non-neoplasm controls
CEA: carcinoembryonic antigen
CA19-9: carbohydrate antigen 19-9
NSCLC: non-small cell lung cancer
ESCC: esophageal squamous cell carcinoma
PBS: phosphate-buffered saline
TEM: transmission electron microscopy
NTA: nanoparticle tracking analysis
FDR: false discovery rate
ROC: receiver operating characteristic curves
AUC: area under the ROC curve
