# Supplementary figures and images for: Loss of Circulating Exosomal miR-92b is a Novel Biomarker of Colorectal Cancer at Early Stage
Source: Int J Med Sci. 2019 Aug 14;16(9):1231–7. doi: 10.7150/ijms.34540 (PMC6775270; doi:10.7150/ijms.34540)

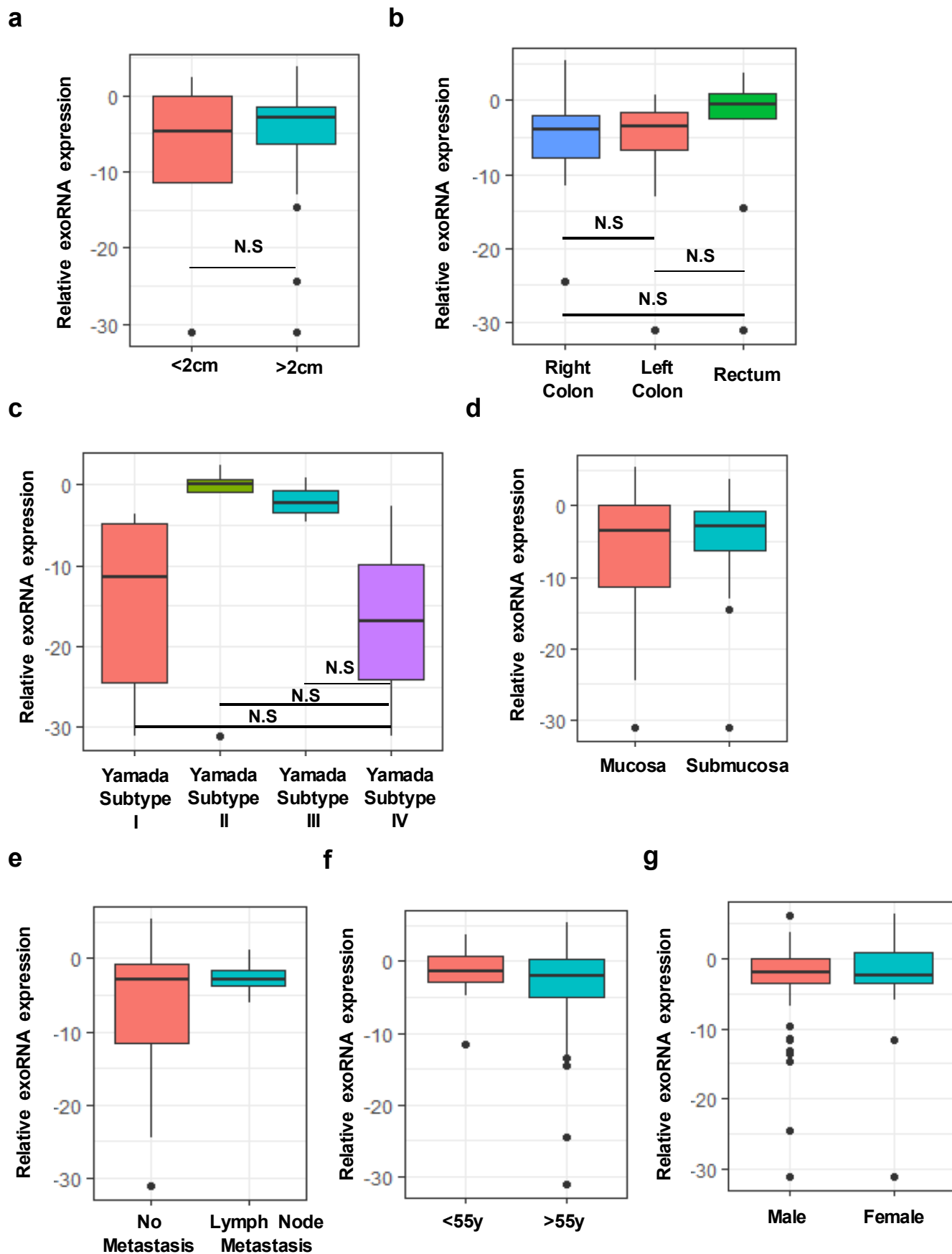

**a**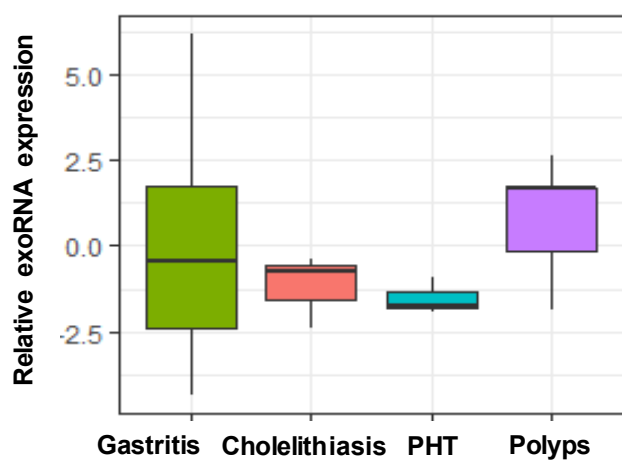**b**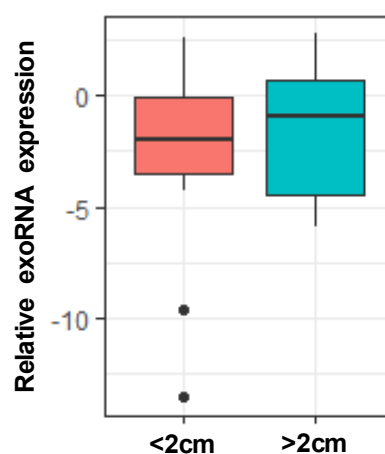**c**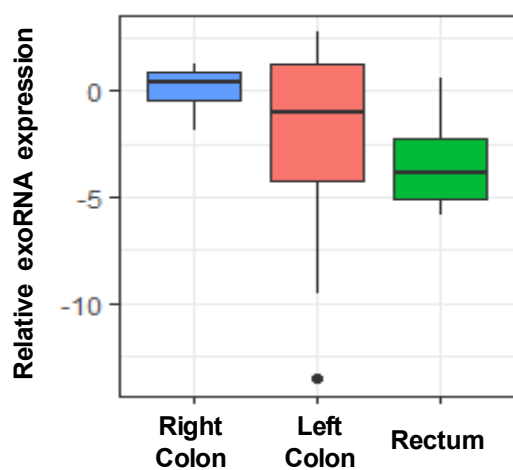**d**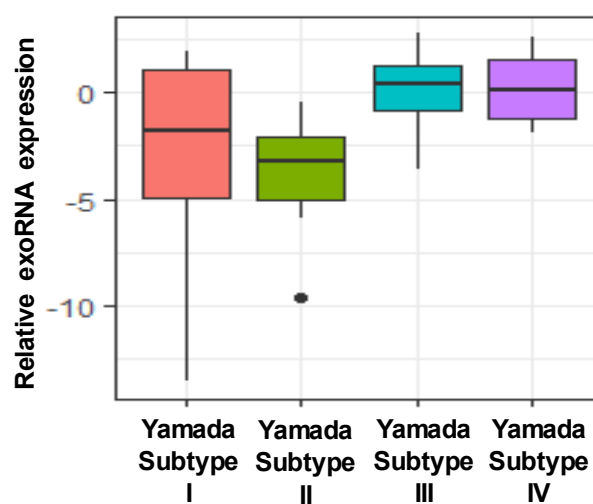**e**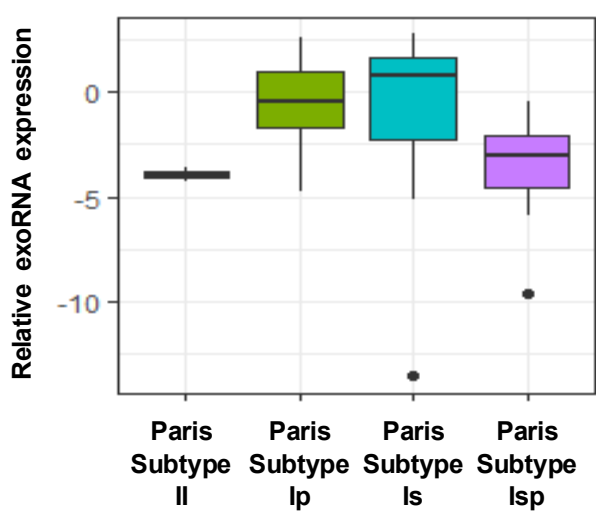

Supplement: Supplementary file 1 — Supplementary figures and tables. [file ijmsv16p1231s1.pdf]
